# Expression of LRRC8/VRAC Currents in *Xenopus* Oocytes: Advantages and Caveats

**DOI:** 10.3390/ijms19030719

**Published:** 2018-03-02

**Authors:** Héctor Gaitán-Peñas, Michael Pusch, Raúl Estévez

**Affiliations:** 1 Facultat de Medicina, Departament de Ciències Fisiològiques, Universitat de Barcelona-IDIBELL, C/Feixa Llarga s/n, L’Hospitalet de Llobregat, 08907 Barcelona, Spain; hektorgp@hotmail.com; 2 Centro de Investigación en red de Enfermedades Raras (CIBERER), Instituto de Salud Carlos III (ISCIII), 08907 Barcelona, Spain; 3 Istituto di Biofisica, Consiglio Nazionale delle Ricerche (CNR), I-16149 Genova, Italy; michael.pusch@ge.ibf.cnr.it

**Keywords:** volume-regulated anion channel, LRRC8, *Xenopus* oocytes, structure-function

## Abstract

Volume-regulated anion channels (VRACs) play a role in controlling cell volume by opening upon cell swelling. Apart from controlling cell volume, their function is important in many other physiological processes, such as transport of metabolites or drugs, and extracellular signal transduction. VRACs are formed by heteromers of the pannexin homologous protein LRRC8A (also named Swell1) with other LRRC8 members (B, C, D, and E). LRRC8 proteins are difficult to study, since they are expressed in all cells of our body, and the channel stoichiometry can be changed by overexpression, resulting in non-functional heteromers. Two different strategies have been developed to overcome this issue: complementation by transient transfection of LRRC8 genome-edited cell lines, and reconstitution in lipid bilayers. Alternatively, we have used *Xenopus* oocytes as a simple system to study LRRC8 proteins. Here, we have reviewed all previous experiments that have been performed with VRAC and LRRC8 proteins in *Xenopus* oocytes. We also discuss future strategies that may be used to perform structure-function analysis of the VRAC in oocytes and other systems, in order to understand its role in controlling multiple physiological functions.

## 1. Identification of LRRC8A as a Component of VRACs

In response to a reduction in extracellular osmolarity (or increase in intracellular osmolarity), water moves following the gradient of osmolarity through aquaporin water channels [1]. This movement of water causes an increase in cell size. In response to this increase, cells activate different regulatory mechanisms in a process called regulatory volume decrease (RVD), involving several transporters and ion channels [2]. Among the latter, volume-regulated anion channels (VRACs) are a major player in volume regulation [3]. VRACs release organic osmolytes, such as taurine and glutamate, and anions such as chloride [4,5,6]. This release changes the driving force of osmolarity and results in an efflux of water, restoring the original cell size.

The identification of proteins mediating VRAC activity was very difficult [7]. First, this plasma membrane channel appears to be expressed in all cells of our body. Therefore, no cell line can be used to find the channel by a strategy of functional expression cloning. Second, the channel is normally closed, but can be activated by cell swelling or by signaling cascades [8]. Thus, one could identify a protein as a VRAC candidate, but in fact, this protein may be activating endogenous VRACs by changing swelling, signal cascades, or other cell biology processes affecting the endogenous VRACs. This may have been the case for several VRAC candidates that ultimately were not VRAC proteins, such as ICln, P-glycoprotein, and even very recently, the ClC-3 chloride/proton antiporter [9].

However, the LRRC8A protein has been identified as a molecular component of the VRAC. As happens many times in science, two groups identified and published their discoveries at the same time using the same strategy [10,11]: a genome-wide RNA interference screen using a fluorescence readout of VRAC activity (using previously developed anion-sensitive yellow fluorescent proteins [12]). Another group performed a similar type of screening in *Drosophila* cells and identified Bestrophin [13], but no LRRC8 proteins, since they are not present in *Drosophila*. siRNA screening has also recently been used to identify a component of the maxi-Cl channel [14], so this strategy or variations of it could be used to identify other unknown chloride channels in wild-type cells or cells deficient for already-known chloride channels. Thus, RNA interference of LRRC8A [10] or knockout of LRRC8A [11,15] diminished or abolished VRAC activity. As LRRC8A showed homology to pannexins [16], which are also able to allow permeation of many ions and molecules, such as ATP [17,18], and which showed overlapping inhibitor pharmacology with VRAC [19,20,21,22], it was logical to propose that LRRC8 was a VRAC component or was needed for VRAC activation. Consistent with LRRC8A being a VRAC component, expressed LRRC8A was localized at the plasma membrane after transient transfection in cell lines [10,11].

## 2. VRAC Is a Heteromer of LRRC8A Plus Another LRRC8 Protein

Once LRRC8A had been identified, the next logical step was to assess whether LRRC8A overexpression in cell lines would enhance VRAC currents. Interestingly, its overexpression reduced VRAC currents [10,11]. This effect was not observed [10] when overexpressing a LRRC8A mutant found in an agammaglobulinemia patient who lacked circulating B cells [23]. Expression of LRRC8A in cells with stable reduced expression of LRRC8A by lentiviral-mediated RNAi or in cells with deleted LRRC8A (by CRISPR-Cas) rescued VRAC activity. These rescue experiments thus demonstrated that LRRC8A was essential for VRAC activity [10,11].

It was hypothesized that VRAC may be a heteromer of different proteins, and thus, overexpression of one component in cell lines may lead to a stoichiometry that could be incompatible with VRAC activity [11]. In fact, immunoprecipitation of endogenous LRRC8A detected LRRC8B, LRRC8C, LRRC8D, and LRRC8D [15]. In heterologous overexpression systems, this interaction was verified for each of the proteins expressed alone [11], and it was verified later (by sequential co-immunoprecipitation) that VRAC heteromers could contain three proteins (LRRC8A, LRRC8C, and LRRC8E) [24]. Furthermore, LRRC8C was not present at the plasma membrane when overexpressed alone, but arrived at the plasma membrane in the presence of LRRC8A [11]. Using crosslinking and mass spectrometry, it was suggested that the heteromer may contain six to eight subunits, and that the heteromers may be a heterogenous mix of combinations of LRRC8A plus different LRRC8 accessory subunits (LRRC8B-D) [15]. Recent results in astrocytes have suggested that some specific combinations may be more abundant than others, although biochemical evidence is lacking [25,26].

Functional evidence that these heteromers form VRAC was obtained after genomic disruption of different LRRC8 proteins individually or in combination, combined with rescue experiments [11,15]. Thus, disrupting *LRRC8B-E* genes abolished VRAC activity, although LRRC8A was still present at the plasma membrane. When disrupting all *LRRC8* genes, VRAC activity could be reconstituted after overexpression of LRRC8A plus another LRRC8 member. Only the transient expression of LRRC8A plus LRRC8B did not rescue VRAC activity in cells knocked out for all *LRRC8* genes, although in cells in which endogenous LRRC8A and LRRC8B were still present (*LRRC8(C*/*D*/*E*)^−/−^), VRAC activity was also still present [11]. The reason for this difference remains unknown. It was observed that different channel properties, such as inactivation kinetics, single-channel conductance, single-channel rectification and even anion or substrate selectivity, depend on the specific subunit combination expressed [11,15]. By performing chimeric experiments between different LRRC8 subunits (LRRC8C and LRRC8E), it was concluded that residues from the first predicted extracellular loop might be important in determining differences in their functional properties [27]. Interestingly, differences were found when comparing VRAC activity in cells that express only two subunits (for instance LRRC8A + LRRC8C) with cells that do not express any *LRRC8* gene (*LRRC8*^−/−^) rescued with these two proteins [27]. It was suggested that these differences could be due to different stoichiometries of LRRC8 complexes in overexpressing cells (which may vary in transient transfection in each cell analyzed) compared with cells expressing the subunits endogenously [27].

These experiments demonstrate that investigating the functional properties of VRAC is very laborious and difficult. All genome-edited cell lines with combinations of LRRC8 proteins (five single, six double without considering the A subunit, four triple without considering the A subunit, quadruple, and quintuple, making about 17 different groups) will need to be generated, and probably in different cell lines. The genomic disruption should ideally be verified by Western blot of the different LRRC8 proteins (five different antibodies at least) [24,28]. It will also be important to take into account that LRRC8 proteins may have different properties depending on their level of expression, requiring the analysis of multiple cells to exclude variations that occur during transient transfection experiments in cells. Furthermore, as LRRC8-VRAC-mediated currents activate slowly by hypotonicity, resulting in non-steady current levels, quantitative analysis of VRAC currents is also difficult. Considering the mechanism of activation, it has not been studied whether hypotonicity induced channel translocation. In fact, studies with the reconstituted proteins in lipid bilayers indicated that reducing ionic strength was enough to activate the channel [15].

## 3. VRAC in *Xenopus* Oocytes

In the same way that all cells possess mechanisms to regulate their volume, amphibian oocytes also contain volume-regulated anion channels that show many similarities in their functional properties and pharmacology with the VRAC observed in mammals [29]. 

*Xenopus* oocytes are commonly used for heterologous expression of membrane proteins involved in ion and solute transport [30]. Stage V–VI *Xenopus* oocytes are obtained by partial ovariectomy under anesthesia [31]. As oocytes are surrounded by enveloping follicular and epithelial cells, these cells are typically removed by treatment with collagenase in zero calcium OR-II or ND-96 solutions. Oocytes can also be isolated by manual defolliculation using forceps, but in this case, the oocytes are only partially surrounded by the follicular envelope. Importantly, volume-sensitive chloride currents can be observed in manually defolliculated (or follicle-enclosed), but not in collagenase-defolliculated oocytes [19,31,32,33]. Thus, it was suggested that the volume-regulated anion channel might be located in the follicular cell membrane [32].

*Xenopus* oocytes present many other types of chloride channels in the plasma membrane. One abundant type is the calcium-activated chloride channel [34], which may have a physiological role in preventing polyspermia (they are not present in *axolotl* oocytes that are polyspermic) and are mediated by TMEM16 proteins [35]. Others are the outwardly rectifying chloride channels and hyperpolarization-activated channels, which have an unknown molecular identity [36]. These channels may appear when oocytes are not in a healthy state, for example, when expressing higher amounts of an exogenous protein (as happened with one of the VRAC candidates, ICln, and other proteins like ClC-6) or when oocytes are exposed to higher temperatures [37]. However, these other chloride channels are not modulated by hypotonicity, and can be differentiated from volume-regulated anion channels.

*Xenopus* oocytes do not possess aquaporins, since their expression could be deleterious when the oocytes are released into water, causing membrane disruption (the expression of aquaporins in oocytes causes loss of membrane integrity after exposure to a hypotonic solution) [38]. Thus, it is logical to think that they also do not have VRAC activity, since they do not need it. Furthermore, the lipid composition of the membrane may also contribute to the low water permeability observed in oocytes. It is possible that follicular cells are important in maintaining the integrity of oocytes when exposed to a hypoosmotic solution, such as water, in which frogs live. It is worth mentioning that although oocytes show low water permeability, the effects of hypotonicity on expressed chloride channels, such as ClC-2, have been examined in oocytes [36]. It should also be remembered that the kinetics of activation by hypotonic stimuli in *Xenopus* oocytes may be slower than in mammalian cells. 

## 4. First Studies of Expression of LRRC8 Protein in *Xenopus* Oocytes

Based on the above information, and considering two important ideas: (1) collagenase-defolliculated oocytes do not have volume-regulated channels, and (2) hypotonicity can activate exogenous chloride channels (such as ClC-2) in collagenase-defolliculated oocytes, we considered the possibility that we could use *Xenopus* oocytes to characterize the VRAC activity induced by human LRRC8 proteins.

**Figure 1:**
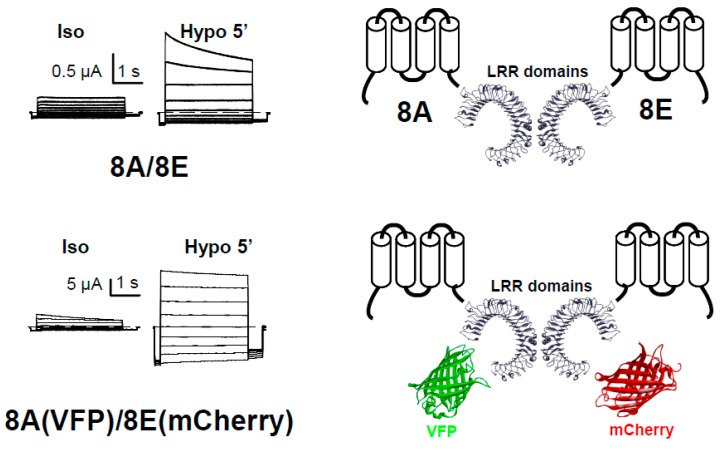
Functional expression of LRRC8-mediated VRAC currents in *Xenopus* oocytes. Currents of single oocytes co-injected with 8A/8E or 8A-VFP/8E-mCherry in response to an iv-pulse protocol in isotonic conditions (Iso) or after a 5 min perfusion with a hypotonic solution (Hypo). On the right, we show the scheme for the injected LRRC8 proteins.

Expression of LRRC8A alone in *Xenopus* oocytes did not induce any significant current in any of our experiments involving isotonic or hypotonic conditions [39]. In contrast, Hammer et al. observed chloride currents co-expressing LRRC8A and AQP1 in hypotonic conditions [40]. As these oocytes swell due to the presence of AQP1, chloride currents may be endogenous. In contrast, we observed clear chloride currents after hypotonic stimuli in oocytes expressing LRRC8A plus LRRC8E (Figure 1) and smaller currents with LRRC8D, but not with LRRC8B or LRRC8C [39]. Although these first experiments were promising, the currents were of small magnitude (about 2 μA) when expressing these untagged human LRRC8 proteins, which made the characterization experiments very difficult. It is worth mentioning that we did not analyze protein expression levels, since we did not have specific antibodies available, and we did not use strategies to boost expression, such as codon optimization, so current levels could still be improved.

As one of our objectives was to study whether the biochemistry and cell biology of LRRC8 proteins expressed in oocytes could be similar to that in cell lines, we fused VFP and mCherry (m stands for monomeric) to the C-terminus of the LRRC8A and LRRC8E proteins, respectively (the nucleotide sequence of these proteins is shown in Figure S1). Unexpectedly, these fluorescently-tagged LRRC8 proteins showed increased activity compared with the untagged proteins (in some cases about 40 μA in hypotonic conditions) and importantly, currents were also observed in isotonic conditions for the tagged proteins (Figure 1) [39]. In hypotonic conditions, the current magnitude was sometimes so high that it caused series resistance problems, resulting in an apparent loss of the inactivating kinetics seen in isotonic conditions. Similar results were obtained when fusing mCherry to LRRC8C and LRRC8D, but not to LRRC8B, and we also observed that the kinetics of the time-dependence of activation varied with the pair of LRRC8 proteins expressed. This latter result strongly suggested that the currents observed were not endogenous to the oocyte, but were rather the result of the activity of the expressed proteins.

Why did the fusion of the fluorescent proteins to the C-terminus activate the LRRC8 proteins? Other experiments provided answers to this question. First, we observed that expressing only one fluorescent protein in one of the partners was sufficient to activate the channel, although larger effects were observed when both subunits were tagged. Interestingly, when LRRC8A and LRRC8E proteins were fused to mCherry, the currents were much higher. In fact, fluorescent proteins that were monomeric were much better at activating the channel. Second, we studied other tags (e.g., three copies of the hemagglutin (HA) tag or three copies of the flag tag), and we did not observe any activation, indicating that the size of the tags may be critical for significant activation. Third, the constitutive activation of the currents by adding fluorescent proteins was also observed when expressing LRRC8 proteins in HEK cells, but currents were very difficult to analyze, due to the low success rate of giga-seal formation. Fourth, the constitutive currents observed when expressing the fluorescently-tagged constructs were reduced when applying a hyperosmotic solution, indicating that the addition of the fluorescent proteins leads to a shift in the osmosensitivity of the channel [39]. Based on these results, we suggested that the fluorescent protein fused at the C-terminus could act as a “foot-in-the-door” mechanism, changing the gating dependence of osmolarity. In this sense, it has been suggested that the mechanism of the LRRC8-homologous pannexin-1 activation also involves the C-terminus of the channel [18,41,42,43].

**Figure 2:**
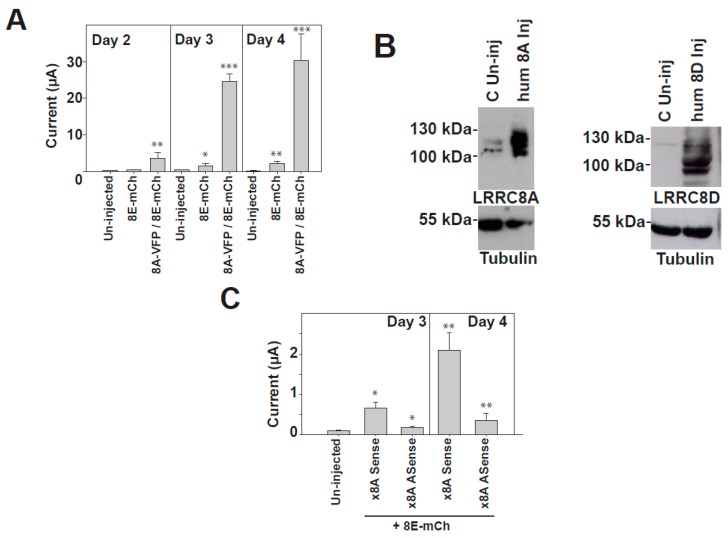
Biochemical and functional analyses of the currents induced by the expression of 8E-mCherry in *Xenopus* oocytes. (**A**) Time course between 2 and 4 days after oocyte injection of the mean values of currents at 60 mV for uninjected oocytes (*n* ≥ 7), oocytes injected with 8E-mCherry (*n* ≥ 18), and oocytes injected with 8A-VFP/8E-mCherry (*n* ≥ 7). Data are from at least two different injections and indicate the mean ± SEM; (**B**) Western blots against some LRRC8 proteins, using β-tubulin as a loading control. We used protein extracts of (from left to right): collagenase-treated uninjected oocytes “C Un-inj” and oocytes injected with 8A or 8D “hum 8A/8D inj”. Three independent Western-blot experiments to detect *Xenopus* LRRC8A and two WB experiments to detect *Xenopus* LRRC8D gave similar results; (**C**) Comparison of the mean values of current at 60 mV between uninjected oocytes (*n* = 4) and oocytes co-expressing 8E-mCherry (20 ng of complimentary ribonucleic acid (cRNA)) plus 10 ng of an oligonucleotide sense “x8A sense” (*n* = 5 at day 3, *n* = 5 at day 4) or plus 10 ng of an oligo antisense “x8A Asense” (*n* = 4 at day 3, *n* = 5 at day 4). Data indicate the mean ± SEM * *p* < 0.05, ** *p* < 0.01, *** *p* < 0.001. Another injection gave similar results.

It is important to consider that although we never observed currents when expressing fluorescently-tagged LRRC8A by itself in *Xenopus* oocytes, we sometimes observed currents when expressing fluorescently-tagged LRRC8 accessory subunits (LRRC8C, D and E) [39]. However, these currents were not always observed (only in about 50% of the batches, and mostly several days after injection, see Figure 2A), and in about 90% of the batches that showed currents, they were smaller than 7.5% of the currents elicited by the co-expression of the two subunits. We thus speculated that although collagenase-digested oocytes do not show VRAC currents, they may contain the *Xenopus* LRRC8A protein. Using an anti-human LRRC8A antibody whose epitope shows about 75% identity with the *Xenopus* LRRC8A protein, we detected protein bands that may represent endogenous forms of glycosylated LRRC8A in collagenase-digested oocytes (Figure 2B). Using antibodies against human LRRC8D, we were unable to observe any protein in collagenase-digested oocytes (Figure 2B). A recent report mentioned in the discussion section indicated that oocytes express LRRC8 proteins [24], but in our hands we could only detect minor amounts of LRRC8A. Therefore, we envisaged that oocytes may contain a small reservoir of maternal LRRC8A protein in anticipation of fertilization, as happens with the potassium channel MinK(IsK)/*Xenopus* KCNQ1 [44], or the heteromeric amino acid transporters rBAT/*Xenopus* b^0,+^AT [45] and 4F2hc/*Xenopus* y^+^LAT-1 [46]. It is thus clear that a minor component of the currents observed when expressing, for instance, LRRC8A-VFP plus LRRC8E-mCherry, may also come from heteromers of *Xenopus* LRRC8A plus LRRC8-mCherry. However, we speculated that this component may be really minor, as double-tagged fluorescent proteins are more active than single-tagged proteins, and the levels of endogenous proteins are lower than those of the exogenous proteins expressed. In order to reduce the amount of endogenous LRRC8A, we performed additional experiments injecting an oligo antisense to *Xenopus* LRRC8A (using the sense oligo as a control), and observed a reduction in the currents elicited when expressing LRRC8E-mCherry alone (Figure 2C). Thus, considering these small caveats, we believe that the expression of the fluorescently-tagged subunits, which are active in isotonic conditions, could be a useful tool to investigate the functional properties of the activity induced by LRRC8 proteins.

## 5. Further Characteristics of the Functional Properties of the Channels Induced by Expression of LRRC8 Proteins in *Xenopus* Oocytes

Taking advantage of the stable constitutive current expressed by fluorescently-tagged LRRC8 proteins in isotonic conditions, it was easier to study different functional properties of the induced currents. For instance, we were able to detect that different LRRC8 heteromers mediate anion and osmolyte flux with subunit-dependent kinetics and selectivity as previously shown in mammalian cells. As another approach to measure substrate permeation through the channel, we measured the influx of radiolabeled substrates, such as taurine, glutamate, and glycine. As some authors have misunderstood these measurements [24], it is important to note here that influx assays can reveal whether a substrate can pass through the channel, but cannot detect differences between different LRRC8 heteromers, as they are performed in non-linear conditions. Importantly, single-channel measurements in the cell-attached configuration revealed that the single-channel current-voltage relationship showed outward rectification [39], in the same manner that has been observed after measuring LRRC8 activity when reconstituted in bilayers [15]. We would like to remark that the swelling of oocytes expressing one aquaporin (AQP1) was reduced when expressing LRRC8 proteins, indicating that as observed in mammalian cells [3], also in oocytes, one can reproduce the functional correlation between VRAC and aquaporins.

Furthermore, we also detected ATP efflux when expressing LRRC8 proteins, this being more evident for LRRC8A/LRRC8E heteromers. It is important to mention that ATP release was only observed at high levels of hypoosmotic swelling. However, these measurements were not performed in voltage clamp conditions, and have not yet been performed in transfected cells. We conclude that further work is needed to confirm that ATP permeates through LRRC8 proteins, although it is clear that it can permeate through their homologous proteins, pannexins [43].

Previously described VRAC inhibitors, such as carbenoxolone (CBX) [22], DCPIB [19], and extracellular ATP [6] blocked currents detected in oocytes expressing LRRC8 proteins. Characterization of the inhibition by CBX suggested that more than one molecule of CBX is required to block the channel (since the Hill coefficient was 1.8), and that it acts from the outside (since it blocked the channel in the pacth clamp outside-out configuration). We also found that LRRC8A/LRRC8E heteromers are dramatically potentiated by oxidation of intracellular cysteine residues by chloramine-T or tert-butyl hydroperoxide [47]. By contrast, LRRC8A/LRRC8C and LRRC8A/LRRC8D heteromers were strongly inhibited by oxidation. The cysteine residues involved in this inhibitory mechanism have not yet been determined. In addition, it has been shown in mammalian cells that unrelated compounds, such as the antibiotic blasticidin [48] and cisplatin drugs [28], can pass through the channel, and the latter is also able to activate the channel [28]. A similar behavior has been observed in oocytes, in which intracellular cisplatin accumulation strongly activates the channel [49]. The mechanism of this activation is unknown, but we speculate that intracellular cisplatin (but not blasticidin) may activate the signal transduction cascades that activate LRRC8 proteins.

## 6. Biochemistry and Cell Biology of LRRC8 Proteins in *Xenopus* Oocytes

Western blot analyses of the fluorescently-tagged proteins revealed that different LRRC8 accessory subunits show different expression levels [39]. In our case, LRRC8E, LRRC8B, and LRRC8C were easily detected, whereas LRRC8D was poorly expressed. It is important to mention here that it has been shown in cells and in oocytes that the expressions of LRRC8A/LRRC8B heteromers do not result in functional activity, although LRRC8B is expressed and detected at the plasma membrane. However, cells expressing only endogenous LRRC8A and LRRC8B (*LRRC8(C/D/E*)**^−^**^/−^) express VRAC currents. Possibly, endogenous but not exogenous LRRC8B may have a posttranslational modification, but evidence is lacking. A recent report associated the expression of LRRC8B in HEK293 cells with the calcium leak in the endoplasmic reticulum [50]. Further work is needed to understand the role of the LRRC8B accessory subunit.

**Figure 3:**
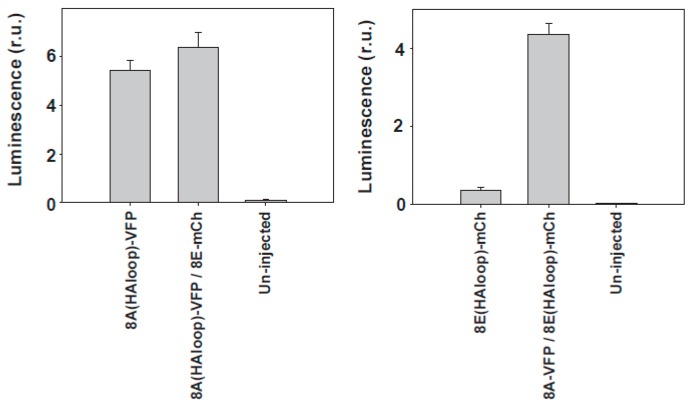
Surface analysis of LRRC8 proteins expressed in *Xenopus* oocytes. Surface expression was examined using a chemiluminescence technique detecting the HA epitope inserted into the first extracellular loop (HAloop) already described [51]. The sequence of LRRC8A with the HA tag was CLPCKW*YPYDVPDYA*VTKDSC and the sequence of LRRC8E with the HA tag was CLPNHE*YPYDVPDYA*LQENLS (HA tag in *italics*). The HA tag did not interfere with the proper function of the proteins. In the panel on the left, we show the membrane levels of 8A-VFP, and in the other panel, the levels of 8E-mCherry. We show here a typical experiment with *n* = 15, 15, and 10 oocytes for the panel on the left, and *n* = 8, 15, and 9 for the panel on the right.

Using TIRF microscopy of the fluorescently-tagged molecules [39], we found that LRRC8A-VFP is expressed at the plasma membrane alone, and that its levels are not increased upon co-expression with LRRC8E-mCherry. By contrast, LRRC8E-mCherry was barely detectable at the plasma membrane, and its levels increased upon co-expression with LRRC8A-VFP. Similar results were recently obtained using extracellular HA-tagged LRRC8A-VFP and LRRC8E-mCherry proteins (Figure 3) expressed in oocytes and detected using ELISA-based luminescence assays. These results are consistent with the results obtained in mammalian cells using immunofluorescence experiments with tagged proteins. Furthermore, using TIRF microscopy in *Xenopus* oocytes, we found that more than two accessory subunits may be associated with LRRC8A in the same complex [39]. Sequential co-immunoprecipitation experiments in mammalian cells with tagged proteins revealed, by an alternative method, that this is the case [24].

As the tagged subunits were relatively immobile in the oocyte membrane, we reasoned that we could use single-step photobleaching to estimate the stoichiometry of the LRRC8 complex [40]. If the heteromers have a fixed stoichiometry, then the distribution of the photobleaching steps would be binomial. However, if the heteromers have a variable (multiple) stoichiometry, then the distribution would be expected to be Poissonian [52]. We observed that the distribution of the photobleaching steps fitted well with a Poisson distribution containing a number of LRRC8 subunits equal or bigger than six, indicating that the stoichiometry is not fixed. Furthermore, when we changed the relative expression of the subunits by increasing the proportion of LRRC8A-VFP, the average number of LRRC8A subunits in the complex also increased, supporting the evidence that the stoichiometry of the heteromers is not fixed, and simply depends on the relative abundance of the subunits. Currents observed when the proportion of LRRC8A-VFP was higher than that of LRRC8E-mCherry were lower compared with the situation where both subunits were expressed in equimolar conditions, suggesting that some of the complexes containing more LRRC8A subunits were not functional, as previously hypothesized [11]. 

## 7. Summary of the Work Performed in *Xenopus* Oocytes and Future Strategies for Structure-Function Analysis of the VRAC

In summary, all of the studies performed to date in collagenase-treated oocytes have demonstrated that this is a valid expression system in which to study LRRC8 proteins, as they have reproduced most of the functional and cell biological properties of LRRC8 proteins expressed in mammalian cells [39]. Furthermore, this expression system has revealed new channel characteristics. All the expression systems have advantages, but also some drawbacks, and thus, all systems are complementary. The fact that some endogenous LRRC8A protein still exists in the oocyte is one of the drawbacks of the *Xenopus* expression system, although the use of antisense oligos and low expression levels may reduce the already small proportion of detected currents. For us, the main advantage of the oocyte expression system is the fact that the channel is constitutively active in physiological conditions, which could facilitate drug screening and structure-function relationships.

One has to acknowledge that studying the VRAC represents a tremendous scientific challenge. Given that there are four accessory subunits, and the fact that expression levels change the properties of the VRAC, as the stoichiometry is variable, studying the properties of a heterologously expressed channel is very difficult. Thus, just simply characterizing a mutant in cell lines is a “tour de force” experiment. Ideally, experiments should first be performed in knockout cell lines for all subunits [11], with control of the expression levels of the subunits by inducible promoters, such as tetracycline-inducible promoters [15]. Changing the expression levels by injecting different amounts of cRNA could be a simpler alternative. However, such studies will always be incomplete, as they will not consider the fact that the channel is formed by multiple subunits. Thus, for each mutant, one should generate a knock-in cell line by CRISP/CAS. In this manner, the mutants will be studied at their endogenous levels. 

The problem of a channel formed by different subunits and with the possibility of having non-functional stoichiometries is not novel, as it has already been observed in nicotinic acetylcholine receptors [53]. Using expression in *Xenopus* oocytes, one solution to overcome this issue has been to create concatenated receptors [54]. However, this approach may be difficult for LRRC8 proteins as the artificial linker could change receptor activation properties, as found with the fusion of GFP proteins. Optimization of shorter linkers may be needed to ensure the expression of the different proteins in only one type of orientation [55]. Alternatively, RNA titration experiments of the different subunits combined with analysis of protein expression should be performed to determine the best cRNA doses to obtain different stoichiometries. In cells, one could use mammalian expression plasmids where the different subunits are separated by viral amino acid sequences, such as the E2A peptide, that has been used to express two subunits of a chloride channel [56]. In this case, one should verify that all proteins show the desirable levels of expression.

Finding inhibitors or activators of the LRRC8 channel could be beneficial for different purposes, such as treating brain ischemia [57] or cancer chemotherapeutics [28]. We think that the oocyte system may be very good for screening of direct activators or inhibitors, because both can be easily measured directly, and because indirect effects on volume-sensing mechanisms can be more easily excluded, if the effect is rapid. This type of screening is more difficult in a cell system where the channels have to be opened first. 

Having a 3D structure of VRAC could be very helpful for finding molecules that bind to the channel, and for understanding the gating mechanism. Unfortunately, the 3D structure of VRAC is not yet known, nor is it known for its homologous pannexin proteins; the structure could be used to understand the structure-function relationship based on its homology. The structures of less related proteins, such as connexins, could also be used in homology modeling experiments [58,59], although their homology to LRRC8 is very low. A model of the C-terminus containing the LRR domains of LRRC8 has already been made using the LRR domains of mouse toll-like receptor 3 [60] (Figure 1). We also envisage that determining the 3D structure of the channel by expressing these proteins heterologously will be a difficult task, as the complex expressed may variably formed by different subunits. Perhaps the structure should be determined by purifying the endogenous source from cells with high VRAC activity, although the amount of protein may be too low. However, the importance of the VRAC in multiple and important biological processes of our body will provide the stimulus for scientists to undercover its fascinating secrets. 

## Supplementary Materials

Supplementary materials can be found at http://www.mdpi.com/1422-0067/19/3/719/s1.
