# Supplementary material for: Expression of LRRC8/VRAC Currents in Xenopus Oocytes: Advantages and Caveats
Source: Int J Mol Sci. 2018 Mar 2;19(3):719. doi: 10.3390/ijms19030719 (PMC5877580; doi:10.3390/ijms19030719)
Supplement: Supplementary file 1 [file ijms-19-00719-s001.pdf]

Figure S1. Nucleotide sequence of the fluorescently tagged LRRC8A-VFP and LRRC8E-mCherry proteins. The LRRC8 isoform, the fluorescent protein and the linker connecting the two proteins are marked with a different color.

hLRRC8A-linker-VFP

accatgattccggtgacagagctccgctactttgcggacacgcagccagcataccggatcctgaagccgtggtgggatgtgttcacagactacatctct
atcgtcatgctgatgattgccgtcttcgggggggacgctgcaggtcacccaagacaagatgatctgcctgccttgtaagtgggtcaccaaggactcctgc
aatgattcgttccggggctgggcagcccctggcccggagcccacctaccccaactccaccattctgccgacccctgacacgggccccacaggcatcaa
gtatgacctggaccggcaccagtacaactacgtggacgctgtgtgctatgagaaccgactgcactggtttgccaagtacttcccctacctggtgcttctg
cacacgctcatcttcctggcctgcagcaacttctggttcaaattcccgcgcaccagctcgaagctggagcactttgtgtctatcctgctgaagtgcttcga
ctcgccctggaccacgagggccctgtcggagacagtggtggaggagagcgaccccaagccggccttcagcaagatgaatgggtccatggacaaaaa
gtcatcgaccgtcagtgaggacgtggaggccaccgtgcccatgctgcagcggaccaagtcacggatcgagcagggtatcgtggaccgctcagagac
gggcgtgctggacaagaaggagggggagcaagccaaggcgctgtttgagaaggtgaagaagttccggacccatgtggaggagggggacattgtgt
accgcctctacatgcggcagaccatcatcaaggtgatcaagttcatcctcatcatctgctacaccgtctactacgtgcacaacatcaagttcgacgtgg
actgcaccgtggacattgagagcctgacgggctaccgcacctaccgctgtgcccacccccctggccacactcttcaagatcctggcgtccttctacatca
gcctagtcatcttctacggcctcatctgcatgtatacactgtggtggatgctacggcgctccctcaagaagtactcgtttgagtcgatccgtgaggagag
cagctacagcgacatccccgacgtcaagaacgacttcgccttcatgctgcacctcattgaccaatacgacccgctctactccaagcgcttcgccgtcttc
ctgtcggaggtgagtgagaacaagctgcggcagctgaacctcaacaacgagtggacgctggacaagctccggcagcggctcaccaagaacgcgca
ggacaagctggagctgcacctgttcatgctcagtggcatccctgacactgtgtttgacctggtggagctggaggtcctcaagctggagctgatccccga
cgtgaccatcccgcccagcattgcccagctcacgggcctcaaggagctgtggctctaccacacagcggccaagattgaagcgcccgcgctggccttcc
tgcgcgagaacctgcgggcgctgcacatcaagttcaccgacatcaaggagatcccgctgtggatctatagcctgaagacactggaggagctgcacct
gacgggcaacctgagcgcggagaacaaccgctacatcgtcatcgacgggctgcgggagctcaaacgcctcaaggtgctgcggctcaagagcaacct
aagcaagctgccacaggtggtcacagatgtgggcgtgcacctgcagaagctgtccatcaacaatgagggcaccaagctcatcgtcctcaacagcctc
aagaagatggcgaacctgactgagctggagctgatccgctgtgacctggagcgcatcccccactccatcttcagcctccacaacctgcaggagattga
cctcaaggacaacaacctcaagaccatcgaggagatcatcagcttccagcacctgcaccgcctcacctgccttaagctgtggtacaaccacatcgcct
acatccccatccagatcggcaacctcaccaacctggagcgcctctacctgaaccgcaacaagatcgagaagatccccacccagctcttctactgccgc
aagctgcgctacctggacctcagccacaacaacctgaccttcctccctgccgacatcggcctcctgcagaacctccagaacctagccatcacggccaa
ccggatcgagacgctccctccggagctcttccagtgccggaagctgcgggccctgcacctgggcaacaacgtgctgcagtcactgccctccagggtgg
gcgagctgaccaacctgacgcagatcgagctgcggggcaaccggctggagtgcctgcctgtggagctgggcgagtgcccactgctcaagcgcagcg
gcttggtggtggaggaggacctgttcaacacactgccacccgaggtgaaggagcggctgtggagggctgacaaggagcaggccacaactttgtatac
aaaagttgtgatgagtaaaggagaagaacttttcactggagttgtcccaattcttgttgaattagatggtgatgttaatgggcacaaattttctgtcagt
ggagagggtgaaggtgatgcaacatacggaaaacttacccttaaattgatttgcactactggaaaactacctgttccatggccaacacttgtcactact
ttgggttatggtctaatgtgctttgctagatacccagatcatatgaaacggcatgactttttcaagagtgccatgcccgaaggttatgtacaggaaaga
actatatttttcaaagatgacgggaactacaagacacgtgctgaagtcaagtttgaaggtgatacccttgttaatagaatcgagttaaaaggtattgat
tttaaagaagatggaaacattcttggacacaaattggaatacaactataactcacacaatgtatacatcactgcagacaaacaaaagaatggaatca
aagctaacttcaaaattagacacaacattgaagatggaggtgttcaactagcagaccattatcaacaaaatactccaattggcgatggccctgtccttt
taccagacaaccattacctgtcctatcaatctgccctttcgaaagatcccaacgaaaagagagaccacatggtccttcttgagtttgtaacagctgctg
ggattacacatggcatggatgaactatacaaatag

LRRC8E-linker-mCh

accatgatcccagtggccgagttcaagcagttcacggaacagcagcctgcgttcaaggtgctcaaaccctggtgggacgtgctggccgagtacctcac
cgtggccatgctcatgattggggtctttggctgcaccctccaggtgacacaggacaagatcatctgtctacccaatcatgagctccaggagaacttatc
agaggccccgtgccagcaattgctgcctcgggggatccctgagcagattggggccctgcaggaggttaaaggccttaagaacaatttggacctgcag
caatacagctttattaaccagctgtgttatgagacggccctgcactggtatgccaagtacttcccttacctcgtggtcattcacacactcatcttcatggt

ctgcaccagtttctggttcaagttccctggcaccagctccaagattgaacacttcatctccatcctgggcaagtgtttcgactctccatggaccaccagg
gccctatccgaggtctccggggagaaccagaagggcccagcagccaccgaacgggctgcggccaccatagtggccatggcagggaccgggccggg
gaaggcaggggagggtgagaaggagaaagtgctggcggaaccggagaaggtggtgaccgagcctccagttgtcaccctgttggacaagaaggagg
gtgagcaagccaaagccctgtttgagaaggtgaagaagttccgcatgcacgtggaagagggcgacatcctgtacaccatgtacatccgacagacggt
gctgaaagtgtgtaagttcctggccatcctggtctacaacctggtctatgtggagaagatcagtttcctggtggcctgtagggtggagacgtcagaggt
cacgggctacgccagcttctgctgcaaccacaccaaggcccacctcttctccaagctggccttctgttacatctcctttgtgtgcatctacggacttacct
gcatctacacgctctactggctcttccaccggcccctcaaggagtactccttccgttccgtgcgggaggagactggcatgggggacattcctgacgtca
agaatgacttcgccttcatgctgcacctcatcgatcagtacgactccctctactccaagcgcttcgccgtcttcctgtccgaggtcagcgaaagccgtct
aaagcagctcaatctcaaccacgagtggacgcccgagaagcttcgacagaagctgcagcgcaatgccgcgggccggctggagctggccctctgcat
gctgccgggtctgcccgacaccgtctttgagctcagtgaggtggagtcactcaggctggaggccatctgcgatatcaccttcccccggggctgtcaca
gctggtgcacttgcaggagctcagcttgctccactcgcccgccaggctacccttctccttgcaggtcttcctgcgggaccacctgaaggtgatgcgcgtc
aaatgcgaggagctccgcgaggtgccgctttgggtgtttgggctgcggggcttggaggagctgcacctggaggggcttttcccccaggagctagctcg
ggcagccaccctggagagcctccgggagctgaagcagctcaaggtgttgtccctccggagcaacgccgggaaggtgccagccagtgtgaccgacgtt
gctggccacctgcagaggctcagcctgcacaacgatggggcccgtctggttgccctgaacagcctcaagaagctggcggcattgcgggagctggagc
tggtggcctgcgggctggagcgcatcccccatgcagtgttcagcctgggtgcgctgcaggaacttgacctcaaggacaaccacctgcgctccatcgag
gaaatcctcagcttccagcactgccggaagctggtcacgctcaggctgtggcacaaccagatcgcctacgtccctgagcacgtgcggaagctcagga
gcctggagcagctctacctcagctacaacaagctggagaccctgccctcccagctcggcctgtgctcaggcctccgtctgctggatgtgtcccacaatg
ggctacactccctgccacccgaggtgggcctcctgcagaacctacagcacctggccctctcctacaatgccctggaggccctgcccgaagagctcttct
tctgccgcaagctgcggacgttgcttctgggcgacaaccagctgagccagctctcgccccacgtgggtgccctcagagccctcagccgcctggagctc
aaaggcaaccgcttagaggcgctgccagaagaacttggcaactgtgggggctcaagaaggcggggctcctggtggaagacacgctttaccagggt
ctgccggcagaagtgcgggacaagatggaggaggaaacaactttgtatacaaaagttgtgatggtgagcaagggcgaggaggataacatggccat
catcaaggagttcatgcgcttcaaggtgcacatggagggctccgtgaacggccacgagttcgagatcgagggcgagggcgagggccgcccctacga
gggcacccagaccgccaagctgaaggtgaccaagggtggcccccctgcccttcgcctgggacatcctgtcccctcagttcatgtacggctccaaggcct
acgtgaagcaccccgccgacatccccgactacttgaagctgtccttccccgagggcttcaagtgggagcgcgtgatgaacttcgaggacggcggcgtg
gtgaccgtgacccaggactcctccctgcaggacggcgagttcatctacaaggtgaagctgcgcggcaccaacttcccctccgacggccccgtaatgca
gaagaagaccatgggctgggaggcctcctccgagcggatgtaccccgaggacggcgccctgaagggcgagatcaagcagaggctgaagctgaagg
acggcggccactacgacgctgaggtcaagaccacctacaaggccaagaagcccgtgcagctgcccggcgcctacaacgtcaacatcaagttggaca
tcacctcccacaacgaggactacaccatcgtggaacagtacgaacgcgccgagggccgccactccaccggcggcatggacgagctgtacaagtag
